# Enantioselective Interactions of Anti-Infective 8-Aminoquinoline Therapeutics with Human Monoamine Oxidases A and B †

**DOI:** 10.3390/ph14050398

**Published:** 2021-04-22

**Authors:** Narayan D. Chaurasiya, Haining Liu, Robert J. Doerksen, N. P. Dhammika Nanayakkara, Larry A. Walker, Babu L. Tekwani

**Affiliations:** 1 Division of Drug Discovery, Department of Infectious Diseases, Southern Research, Birmingham, AL 35205, USA; 2 Department of Bio-Molecular Sciences, School of Pharmacy, University of Mississippi, Oxford, MS 38677, USA; hainingliu14@gmail.com (H.L.); rjd@olemiss.edu (R.J.D.); 3 National Center for Natural Products Research, School of Pharmacy, The University of Mississippi, Oxford, MS 38677, USA; dhammika@olemiss.edu (N.P.D.N.); lwalker@olemiss.edu (L.A.W.)

**Keywords:** 8-Aminoquinolines, primaquine, enantiomers, monoamine oxidase, anti-infective, malaria, antimalarial, leishmaniasis, pneumocystis pneumonia

## Abstract

8-Aminoquinolines (8-AQs) are an important class of anti-infective therapeutics. The monoamine oxidases (MAOs) play a key role in metabolism of 8-AQs. A major role for MAO-A in metabolism of primaquine (PQ), the prototypical 8-AQ antimalarial, has been demonstrated. These investigations were further extended to characterize the enantioselective interactions of PQ and NPC1161 (8-[(4-amino-1-methylbutyl) amino]-5-[3, 4-dichlorophenoxy]-6-methoxy-4-methylquinoline) with human MAO-A and -B. NPC1161B, the (*R*)-(−) enantiomer with outstanding potential for malaria radical cure, treatment of visceral leishmaniasis and pneumocystis pneumonia infections is poised for clinical development. PQ showed moderate inhibition of human MAO-A and -B. Racemic PQ and (*R*)-(−)-PQ both showed marginally greater (1.2- and 1.6-fold, respectively) inhibition of MAO-A as compared to MAO-B. However, (*S*)-(+)-PQ showed a reverse selectivity with greater inhibition of MAO-B than MAO-A. Racemic NPC1161 was a strong inhibitor of MAOs with 3.7-fold selectivity against MAO-B compared to MAO-A. The (*S*)-(+) enantiomer (NPC1161A) was a better inhibitor of MAO-A and -B compared to the (*R*)-(−) enantiomer (NPC1161B), with more than 10-fold selectivity for inhibition of MAO-B over MAO-A. The enantioselective interaction of NPC1161 and strong binding of NPC1161A with MAO-B was further confirmed by enzyme-inhibitor binding and computational docking analyses. Differential interactions of PQ and NPC1161 enantiomers with human MAOs may contribute to the enantioselective pharmacodynamics and toxicity of anti-infective 8-AQs therapeutics.

## 1. Introduction

The 8-aminoquinolines (8-AQ) are an important class of anti-infective therapeutics for treatment, radical cure and prophylaxis of *Plasmodium vivax* malaria [1,2] and for prophylaxis as well as transmission blocking of *Plasmodium falciparum* [3,4]. The utility of 8-AQs has also been suggested for treatment of other protozoal infections [5] and also *Pneumocystis* pneumonia [6,7], an opportunistic infection in AIDS and other immuno-compromised patients [8]. Primaquine hybrid analogs have also shown promising antimycobacterial activity in experimental models [9,10]. Metabolism, pharmacokinetic and pharmacodynamic factors have been implicated in both the anti-infective efficacy [5] and toxicity of 8-AQs [9,10]. The most problematic toxicity is the potential for hemolytic toxicity in glucose 6-phosphate dehydrogenase (G6PD)-deficient individuals [11,12,13,14,15].

**Figure 1:**
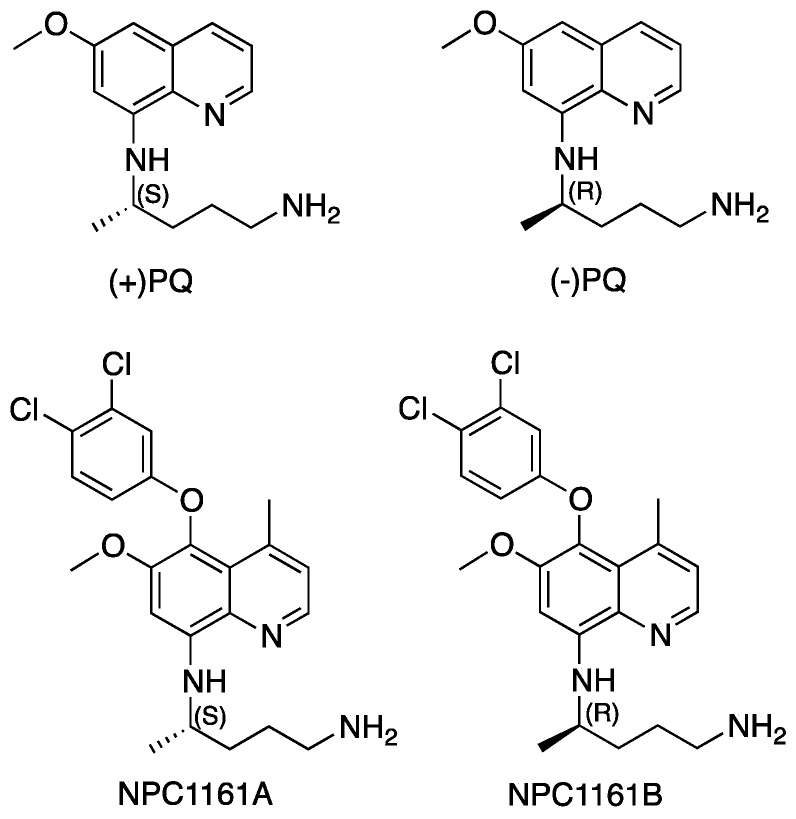
Structures of primaquine (PQ) and NPC1161 enantiomers.

Primaquine (PQ) has broad therapeutic utility for treatment, prophylaxis, radical cure and prevention of transmission of malaria [3]. Mass drug administration of PQ has been suggested for clearance of residual gametocytemia in malaria endemic regions for malaria control [16,17,18]. However, hemolytic toxicity in individuals with genetic deficiency of G6PD limits the use of PQ for malaria radical cure and prevents widespread use of this drug in public health [19,20,21]. PQ is metabolized through both CYP P_450_ and non-CYP mediated pathways [15,22,23,24]. Carboxyprimaquine (carboxy-PQ), generated through the monoamine oxidase (MAO)-dependent oxidative deamination pathway, is a major plasma metabolite of PQ [25,26,27,28]. Rapid metabolism of PQ, including by MAO pathways, results in a short half-life of this drug, and has generally required a long (14 day) treatment regimen for malaria radical cure [29,30,31]. The carboxy metabolite of PQ has been deemed an inactive metabolite [26,32]. New 8-AQ analogs have been developed to improve efficacy and reduce hemolytic toxicity in G6PD-deficient individuals [2,5,33]. Resistance to biotransformation to the MAO-catalyzed carboxy metabolite was employed as an important approach for preparation of metabolically stable 8-AQ analogs [6,34,35,36]. NPC1161, 8-[(4-amino-1-methylbutyl)-amino]-6-methoxy-4-methyl-5-[3,4-dichlorophenoxy]-quinoline (Figure 1), has been identified as a highly efficacious 8-AQ analog in animal models of malaria, *Pneumocystis* pneumonia and visceral leishmaniasis [36,37]. NPC1161B and potentially its metabolites were also reported as transmission-blocking candidates for the treatment of *P. falciparum* [13]. NPC1161 showed several-fold better efficacy compared to PQ and was much superior to tafenoquine (also an 8-AQ antimalarial drug) in rodent malaria models [36]. Tafenoquine, though recently approved for malaria radical cure and prophylaxis, still suffers from significant liability and is not recommended for G6PD-deficient individuals. The racemic 8-AQ analog NPC1161 was resolved into its individual enantiomers (Figure 1). The enantiomers of NPC1161 showed marked differences in their anti-infective efficacy and toxicity in animal models. The (*R*)-(−) enantiomer NPC1161B was found to be several-fold more active than the (*S*)-(+) enantiomer NPC1161A. In addition, the (*R*)-(−) enantiomer showed markedly reduced general toxicity in mice and reduced hematotoxicity in the dog model of methemoglobinemia [36,38]. The enantiomers of PQ also showed marked differences in their anti-infective efficacy and toxicity profiles [39,40]. However, the enantioselective pharmacological profile of PQ in rodents was reversed when compared to NPC1161. The (*S*)-(+)-PQ was found to be more efficacious in mice compared to (*R*)-(−)-PQ. The (*S*)-(+)-PQ also showed higher general toxicity compared to (*R*)-(−)-PQ [41]. (*R*)-(−)-PQ showed rapid metabolism to carboxy-PQ in in vitro studies with primary human hepatocytes [42] and recombinant human MAO enzyme [43,44], while (*S*)-(+)-PQ was relatively resistant to metabolism through this pathway. Rapid metabolism of (*R*)-(−)-PQ through MAO-mediated pathway was also confirmed in in vivo pharmacokinetic studies in rodents [32], non-human primates [41] and healthy human volunteers [45,46]. These studies have led us to clinical evaluation of PQ enantiomers in human volunteers (NCT04073953; NCT03934450; NCT02898779), and NPC1161B is being advanced to clinical evaluation as an enantiomerically pure 8-AQ.

These studies suggested enantioselective interactions of 8-AQ anti-infective therapeutics PQ and NPC1161 with MAO enzymes. The differential roles of MAO-A and -B isoforms in the oxidative deamination of endogenous biogenic amines and regulation of levels of neurotransmitter amines in the neuronal tissues have been studied in detail [47,48,49]. However, contributions of amine oxidases to the metabolism of xenobiotics and therapeutic drugs have received less attention [50]. Although the majority of drugs are metabolized through CYP P_450_ pathways, non-CYP P_450_ mediated pathways also significantly contribute to the metabolism of drugs and xenobiotics [50,51,52]. Amine oxidases of different classes are distributed in all tissues, and are abundant in plasma as well. Amine oxidases catalyze oxidative metabolism of drugs with primary and secondary amine groups [52]. Involvement of MAOs in the in vivo metabolism of several clinically used drugs has been demonstrated [53,54,55,56]. A key role for MAO in the metabolism of primaquine and the generation of carboxy-PQ, a major plasma metabolite, has been demonstrated [25,27,57]. Blocking of the terminal amino group on the 8-AQ side chain protected it from MAO-mediated metabolism, but greatly compromised the efficacy [58]. Primaquine was identified as both a substrate and an inhibitor for monoamine oxidase A [25,44,58,59], while NPC1161 inhibited MAO-A and -B with significantly higher potency compared to primaquine [59]. These studies were further extended to investigate differential interactions of individual PQ and NPC1161 enantiomers (Figure 1) with recombinant human MAO-A and -B. Stereoselective recognition of substrates and differential inhibition of MAO-A and -B with enantiomers have been previously established [60,61,62].

The enantiomers were tested for dose-dependent inhibition of MAO-A and -B at steady-state substrate concentration to determine IC_50_ values. Binding and interactions of 8-AQ enantiomers with human MAO-A and -B were also determined by kinetics of enzyme activity inhibition at varying substrate concentrations, analysis of enzyme-inhibitor complex formation by equilibrium dialysis, and computational analysis of enzyme-inhibitor binding and interactions.

## 2. Results

### 2.1. Determination of Inhibitory Effects of 8-AQs Analogs on MAO-A and -B

**Figure 2:**
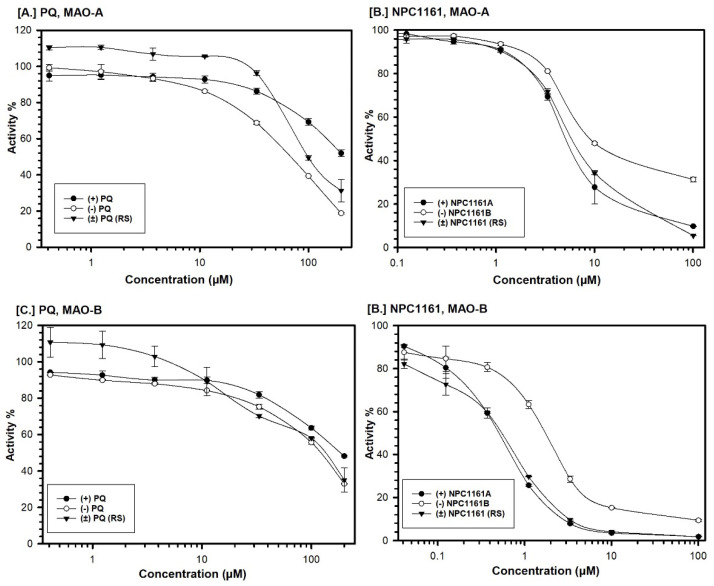
Concentration-dependent inhibition profile of MAO-A (**A**,**B**) and –B (**C**,**D**) with primaquine, NPC1161 and their enantiomers. Each point represents the values mean ± SD of at least three observations.

PQ and NPC1161 (racemic mixtures and pure enantiomers) were tested for inhibition of human MAO-A and -B at 7 concentrations of the inhibitor at steady-state enzyme kinetics with fixed saturating concentration of the substrate. IC_50_ values were computed from the dose-response enzyme activity inhibition profiles (Figure 2). PQ and its enantiomers showed moderate inhibition of MAO-A and -B (Figure 2A,C and Table 1). NPC1161 and its enantiomers showed strong and selective inhibition of MAO-B as compared to MAO-A (Figure 2B,D and Table 1). Racemic PQ showed only 1.2-fold selectivity for inhibition of MAO-A versus MAO-B. Overall, (*R*)-(−)-PQ was a stronger inhibitor of MAO-A and -B compared to (*S*)-(+)-PQ. (*S*)-(+)-PQ was a less-potent inhibitor of MAO-A and -B compared to racemic PQ and (*R*)-(−)-PQ. (*S*)-(+)-PQ showed selective inhibition of MAO-B versus -A, while (*R*)-(−)-PQ showed about 1.5-fold selectivity for inhibition of MAO-A vs MAO-B (Table 1).

The racemic mixture and both enantiomers of NPC1161 showed selective inhibition of MAO-B versus -A. NPC1161A, the (*S*)-(+) enantiomer, was a strong inhibitor of MAO-B with an IC_50_ value of 540 nM and almost 10-fold selectivity compared to MAO-A (IC_50_ 5.24 μM). NPC1161B, the (*R*)-(−) enantiomer, showed moderate inhibition of MAO-A (IC_50_ 9.7 μM) and about 3.3-fold more potent inhibition of MAO-B (IC_50_ 2.93 μM) (Table 1). The enantiomers of NPC1161 (NPC1161A and NPC1161B) were further evaluated for selectivity of binding and interaction with human MAO-A and -B.

### 2.2. Enzyme-Inhibition Kinetics for Interaction of 8-AQs with MAO-A and -B

**Figure 3:**
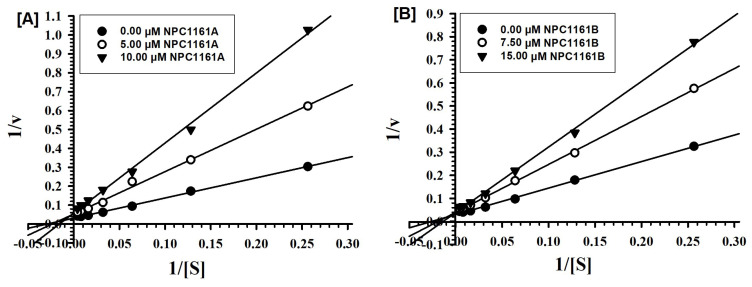
Kinetic characteristics of inhibition of recombinant human MAO-A with (**A**) (*S*)-(+)-NPC1161A and (**B**) (*R*)-(−)-NPC1161B; V = nmoles/min/mg protein and S = substrate kynuramine concentration (μM).

The individual enantiomers (*S*)-(+)-NPC1161A and (*R*)-(−)-NPC1161B were tested against MAO-A and -B at varying concentrations of the substrate (kynuramine, a nonselective substrate), to investigate the nature of the enzyme inhibition. Based on dose–response inhibition, at least two concentrations of the inhibitors were selected, one below and another above the IC_50_ value for the inhibition-kinetic studies. For each experiment, three sets of assays were done at varying concentrations of the substrate, one control without inhibitors, and the other two at fixed concentrations of the inhibitor. The data were analyzed by double-reciprocal Lineweaver–Burk plots to determine Ki (inhibition/binding affinity) values. Binding of (*S*)-(+)-NPC1161A and (*R*)-(−)-NPC1161B with human MAO-A affected Km (affinity of the substrate for the enzyme) as well as Vmax (maximum enzyme activity), which indicated that the inhibition of MAO-A with NPC1161 enantiomers was non-competitive (Figure 3A,B).

**Figure 4:**
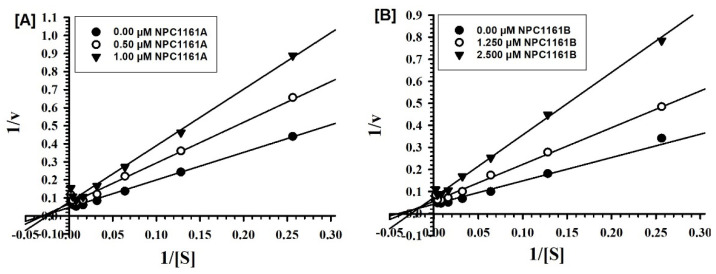
Kinetic characteristics of inhibition of recombinant human MAO-B with (**A**) (*S*)-(+)-NPC1161A and (**B**) (*R*)-(−)-NPC1161B; V = nmoles/min/mg protein and S = substrate kynuramine concentration (μM).

Similarly, the inhibition of MAO-B by (*S*)-(+)-NPC1161A and (*R*)-(−)-NPC1161B was also non-competitive (Figure 4A,B).

Ki values were computed from the double reciprocal plots (Table 2). The affinity for binding of (*S*)-(+)-NPC1161A with MAO-B was higher as compared to the (*R*)-(−)-NPC1161B (Ki value 0.294 μM vs 1.385 μM). However, (*S*)-(+)-NPC1161A and (*R*)-(−)-NPC1161B showed similar, moderate but significant affinity for binding with human MAO-A (Figure 4A,B and Table 2).

### 2.3. Enzyme-Inhibition Kinetics for Interaction of 8-AQs with MAO-A and -B

The characteristics of binding of (*S*)-(+)-NPC1161A and (*R*)-(−)-NPC1161B with MAO-A and -B were also evaluated by analysis of the dissociation of the enzyme-inhibitor complex using equilibrium dialysis. Recombinant human MAO-A and -B were incubated with high concentrations of the inhibitors NPC1161A and NPC1161B to facilitate formation of enzyme-inhibitor complex. The enzyme-inhibitor complex mixtures were dialyzed overnight against the buffer solution with low salt concentration. Activity of the enzymes was measured in these preparations before and after the dialysis.

**Figure 5:**
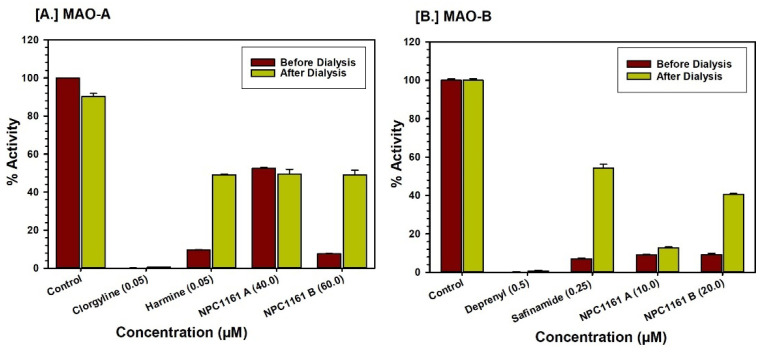
Evaluation of binding of (**A**) (*S*)-(+)-NPC1161A and (**B**) (*R*)-(−)-NPC1161B with recombinant human enzymes. The recovery of MAO-A and -B catalytic activity of the enzyme after equilibrium dialysis is plotted. Each bar shows mean ± SD of triplicate values.

Analysis of the MAO-A without any inhibitors showed about 20% inactivation of the enzyme, as indicated by enzymatic activity of the rhMAO-A before and after dialysis (Figure 5). Incubation of MAO-A with (*S*)-(+)-NPC1161A (40 μM) caused more than 49% inhibition of the enzyme catalytic activity, inhibition which could not be reversed after dialysis. Incubation of MAO-A with 60 μM of (*R*)-(−)-NPC1161B caused more than 92% inhibition of the enzyme catalytic activity, which could be partially reversed after dialysis. This observation showed recovery of the enzyme catalytic activity due to partial dissociation of MAO-A-NPC1161B complex. Thus, binding of (*S*)-(+)-NPC1161A with MAO-A was irreversible and (*R*)-(−)-NPC1161B with MAO-A was partially reversible (Figure 5A).

Similarly, incubation of (*S*)-(+)-NPC1161A (10 μM) with MAO-B produced 90% inhibition of the enzyme activity, which was only marginally reversed after dialysis (13%). Incubation of MAO-B with (*R*)-(−)-NPC1161B (20 μM) inhibited the catalytic activity of the enzyme by 90%, which was significantly recovered (40%) after dialysis of the MAO-B-NPC1161B incubation mixture (Figure 5B). The binding assay results demonstrate reversible binding of (*R*)-(−)-NPC1161B with MAO-B.

### 2.4. Computational Analysis of Binding of (S)-(+)-PQ, (R)-(−)-PQ, (S)-(+)-NPC1161A and (R)-(−)-NPC1161B with MAO-A and MAO-B

**Figure 6:**
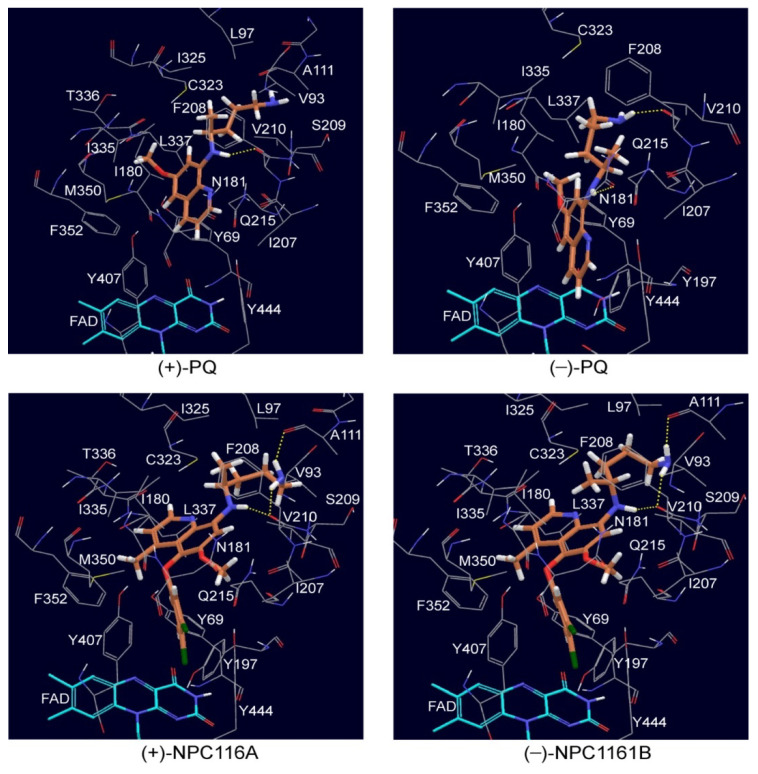
The preferred binding poses of (*S*)-(+)-PQ, (*R*)-(−)-PQ, (*S*)-(+)-NPC1161A and (*R*)-(−)-NPC1161B with MAO-A.

The preferred binding poses of (*S*)-(+)-PQ, (*R*)-(−)-PQ, (*S*)-(+)-NPC1161A and (*R*)-(−)-NPC1161B with MAO-A are shown in Figure 6. For (*S*)-(+)-PQ, the 8-NH group forms a hydrogen bond with the backbone carbonyl oxygen of F208 with an 8-NH_(*S*)-(+)-PQ_…O=C_F208_ distance of 1.85 Å. In addition, (*S*)-(+)-PQ further interacts with several hydrophobic residues, including F352, M350, I180, L337, I335, I325, L97, V93, A111, V210, F208 and I207. In contrast, (*R*)-(−)-PQ binds in a different position that is much closer to FAD (see Figure 6). Specifically, its aminoquinoline ring forms a π–π stacking interaction with Y407. In addition, the 8-NH and terminal –NH_2_ groups form hydrogen bonds (2.33 and 2.04 Å, respectively) with N181 and the backbone of F208, respectively. In this binding pose, the asymmetric carbon of (*R*)-(−)-PQ is close to Q215 (see Figure 6), with the distance between this carbon and the terminal nitrogen of Q215 of 3.18 Å. Hence, only a hydrogen atom can comfortably fit between the C and N, so it is not surprising that a similar binding pose was not favorable for (*S*)-(+)-PQ. Furthermore, the docking scores (Table 3) suggest that (*R*)-(−)-PQ should bind more strongly than (*S*)-(+)-PQ. This matches with the experimental results that (*R*)-(−)-PQ has a smaller IC_50_ than (*S*)-(+)-PQ.

(*S*)-(+)-NPC1161A and (*R*)-(−)-NPC1161B bind in a similar manner to MAO-A, with the major differences lying in the orientation of the substituents connected to the asymmetric carbon. They both form hydrogen bonds with the backbones of A111 and F208 via the terminal –NH_2_ and the 8-NH group, respectively. This makes the 5-phenoxy substituent face towards FAD. No orientations with the terminal –NH_2_ lying close to FAD were observed in the top 10 binding poses. Hence, this may explain the fact that they are not good substrates for MAO-A. In addition, the docking scores (Table 3) also indicate that (*S*)-(+)-NPC1161A and (*R*)-(−)-NPC1161B should bind more strongly than (*S*)-(+)-PQ and (*R*)-(−)-PQ into MAO-A, consistent with experimentally measured IC_50_ values.

The most preferred binding poses of (*S*)-(+)-PQ, (*R*)-(−)-PQ, (*S*)-(+)-NPC1161A and (*R*)-(−)-NPC1161B with MAO-B are shown in Figure 6. (*S*)-(+)-PQ and (*R*)-(−)-PQ bind in a similar manner, with the major differences lying in the orientation of the substituents connected to the asymmetric carbon. This is different from their binding mode in MAO-A, perhaps due to the larger volume of the MAO-B binding pocket (~700 Å^3^) compared to that of MAO-A (~550 Å^3^) [64]. Both (*S*)-(+)-PQ and (*R*)-(−)-PQ form hydrogen bonds with Y326 and the backbone carbonyl oxygen of I199 via their terminal –NH_2_ group. In addition, they also interact with several hydrophobic residues including F343, M341, L171, L328, F168, I316, I199 and I198. Furthermore, the docking scores suggest that (*R*)-(−)-PQ should bind more strongly than (*S*)-(+)-PQ in MAO-B, which matches with experimentally measured IC_50_ values.

**Figure 7:**
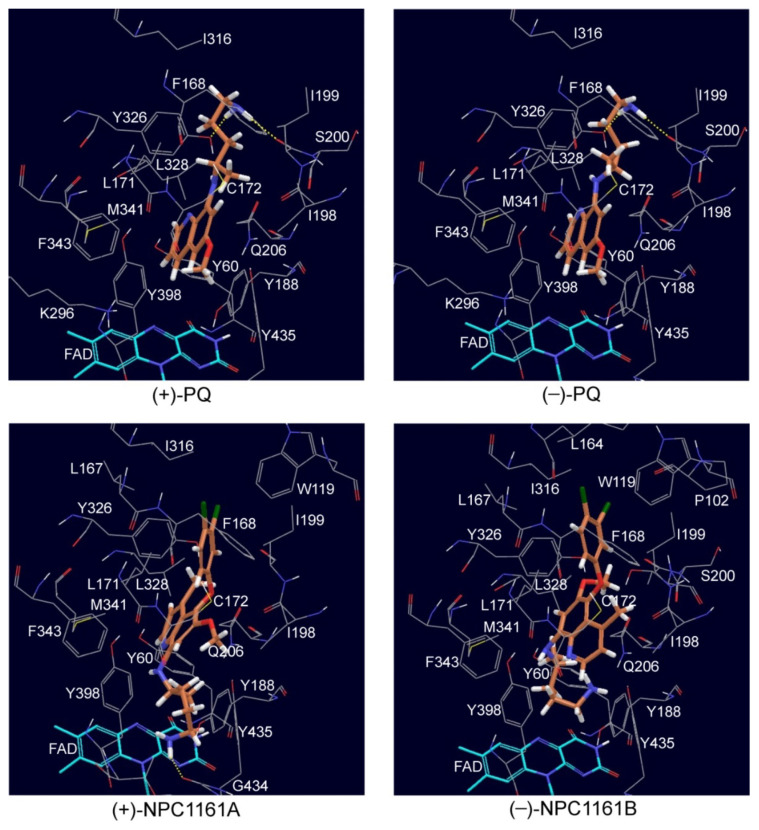
The preferred binding poses of (*S*)-(+)-PQ, (*R*)-(−)-PQ, (*S*)-(+)-NPQ1161A and (*R*)-(−)-NPC1161B with MAO-B.

The binding orientations of (*S*)-(+)-NPC116A and (*R*)-(−)-NPC1161B with MAO-B are slightly different. However, they each have their terminal alkyl group facing toward FAD (see Figure 7), which is not found in their binding modes with MAO-A. No hydrogen bonds were observed in these two binding poses. Hence, hydrophobic interactions make the largest contribution to the binding. (*S*)-(+)-NPC1161A has the most negative docking scores (binds best), which is consistent with its lowest experimentally measured IC_50_. Furthermore, the docking scores also match with other experimentally observed features, including the preferred binding of (*S*)-(+)-NPC1161A with MAO-B over MAO-A and the greater inhibition of MAO-B by (*S*)-(+)-NPC1161A than by (*S*)-(+)-PQ.

## 3. Discussion

The results presented here confirm enantioselective interactions of 8-AQ anti-infective drugs PQ and NPC1161 with human MAO-A and -B. The differential roles of MAO-A and -B isoforms in the oxidative deamination of endogenous biogenic amines and regulation of levels of neurotransmitter amines in neuronal tissues have been studied in detail [47,48,49]. However, contributions of MAOs to the metabolism of therapeutic drugs and their pharmacokinetic/pharmacodynamics-linked pharmacological implications have received less attention [50]. The involvement of MAOs in the metabolism of therapeutic drugs has been demonstrated in laboratory animals as well as in vitro systems, such as animal or human hepatocytes or rat liver fractions [50]. The results from this study show preferable interaction of (*R*)-(−)-PQ with MAO-A compared to that with MAO-B, as reflected by relative Ki values and the stronger binding indicated in the computational docking studies. These observations support earlier in vitro observations [58] and also more rapid metabolism of (*R*)-(−)-PQ compared to (*S*)-(*+*)-PQ through MAO-A mediated pathways to carboxy-PQ in vitro [42,43], as well as in vivo in laboratory animals and clinical studies in healthy human volunteers [32,38,41,45]. In vitro experiments with primary human hepatocytes and recombinant human enzyme confirmed the role of MAO-A in the metabolism of (*R*)-(−)-PQ to carboxy-PQ [25,44,65]. A recent clinical study on PQ metabolism with allelic and genotypic analysis in healthy human volunteers suggested that polymorphisms in MAO-A (rs6323, 891G>T) significantly influenced primaquine metabolism [27]. This study confirmed the role of MAO-A in the metabolism of PQ. Both in vitro and in vivo studies have suggested negligible metabolism of (*S*)-(+)-PQ to the corresponding carboxy-PQ metabolite through the monoamine oxidase-mediated pathway [32,38,41,42,43,45]. The lack of inhibition of MAO-A observed here, even up to 200 μM of (*S*)-(+)-PQ, confirms these results. The 5-phenoxy 8-AQ analogs have shown significantly improved activity for malaria radical cure, and also resistance to metabolism of their side-chain terminal amine through the MAO-mediated pathway. Tafenoquine (4-N-[2,6-dimethoxy-4-methyl-5-[3-(trifluoromethyl)phenoxy]quinolin-8-yl]pentane-1,4-diamine) has been recently approved by the US FDA as a single dose *P. vivax* radical cure and prophylaxis against *P. falciparum* [66,67]. However, the use of tafenoquine is still contraindicated in G6PD deficiency [68]. Both PQ and tafenoquine are used as racemic forms. The enantioselective pharmacologic profile of PQ and NPC1161 has been extensively described now in in vitro and in vivo preclinical models. Previous studies have established a better therapeutic profile of NPC1161B (the R enantiomer) compared to NPC1161A (the S enantiomer). The enantiomers of NPC1161 have been evaluated in experimental animal models of malaria, *Pneumocystis carinii* pneumonia, and visceral leishmaniasis infections. The individual enantiomers of NPC1161 have also been evaluated for propensity to elicit methemoglobinemia in beagle dogs. The (R) (-)-enantiomer NPC1161B was found to be several-fold more active than the (S) (+)-enantiomer NPC1161A in all of these animal models of infection. In addition, the (-) enantiomer showed markedly reduced general toxicity in mice and reduced toxicity in the dog model of methemoglobinemia [34]. NPC1161B is being considered for clinical advancement for malaria radical cure in enantiomerically pure form. However, differential pharmacological properties of tafenoquine enantiomers have not been reported. NPC1161 and its enantiomers have shown relative resistance to metabolism to the corresponding carboxy metabolite through MAO-mediated pathways. Further, computational docking studies support these results. The docking of PQ and NPC1161 enantiomers with MAO-A and -B was investigated using the Glide XP docking approach. The trend of the obtained docking scores matches very well with the experimentally measured IC_50_s. For example, the NPC1161 enantiomers have more negative docking scores than their PQ counterparts. This is presumably because of a strong hydrophobic interaction with the protein by the 5-phenoxy substituent of NPC1161 enantiomers. In addition, the binding poses of the NPC1161 enantiomers with MAO-A show that the 5-phenoxy substituents face towards FAD, which can explain why they are not good MAO-A substrates, as observed experimentally. Furthermore, the second-ranked binding poses of the PQ enantiomers with MAO-A explain why (*R*)-(−)-PQ is a better MAO-A substrate than (*S*)-(+)-PQ, since the terminal –NH_2_ and the α-CH_2_ groups of (*R*)-(−)-PQ are closer to the N5 of FAD.

Tafenoquine tested in racemic form also showed selective inhibition of human MAO-B (IC_50_ 0.95 ± 0.07 μM) compared to MAO-A (IC_50_ 2.70 ± 0.14 μM). Differential interactions of PQ and NPC1161 enantiomers with human MAO-A and -B may be partly responsible for the enantioselective pharmacokinetic, pharmacodynamic, pharmacological and toxicological properties of these anti-infective 8-AQs. Selective and potent inhibition of MAO-B by NPC1161A may also have potential neuropharmacological implications. These studies provide additional justification, in addition to the better efficacy and lower toxicity previously reported, for consideration of clinical advancement of NPC1161B [34], which shows relatively moderate interaction with human MAO enzymes compared to NPC1161A.

## 4. Materials and Methods

### 4.1. Reagents and Chemicals

Recombinant human monoamine oxidase (*r*hMAO)-A and MAO-B were procured from BD Biosciences (Bedford, MA, USA). Kynuramine, a common substrate for MAO-A and -B [69] (obtained as the bromide salt), 4-hydroxyquinoline (the oxidative product of kynuramine) and DMSO were purchased from Sigma (St. Louis, MO, USA). Primaquine and NPC1161 enantiomers were prepared and their enantiomeric purities were checked by NMR and LC–MS/MS analysis with chiral columns as reported earlier [36,38]. Other reagents and chemicals used were of certified high-purity grades procured from reputable commercial vendors. Primaquine and enantiomers were prepared as diphosphate salts, and the NPC1161 and enantiomers as the hemisuccinates.

### 4.2. In Vitro MAO Inhibition Assay

The in vitro enzyme-inhibition assay was designed to measure the effect of primaquine and NPC1161 enantiomers on catalytic activity of *r*hMAO-A and -B. The kynuramine oxidative deamination assay was performed in white flat-bottom 96-well plates as previously described, with minor modifications [63,70]. A fixed concentration of kynuramine substrate and varying concentrations of the test compounds were used to determine the IC_50_ values. Kynuramine concentrations for MAO-A and -B assays were 80 μM and 50 μM, respectively. The concentrations of PQ and NPC1161 enantiomers varied from 0.1 μM to 200 μM, for the rhMAO-A and -B enzyme activity inhibition. The test compounds were dissolved in DMSO, diluted in the buffer solution just before the assay, and pre-incubated with the enzyme (MAO-A (5 μg/mL) or -B (12.5 μg/mL)) for 10 min at 37 °C. The final concentration of DMSO in the enzyme–assay reaction mixtures (total volume 200 μL) did not exceed 1%. The enzymatic reactions were initiated by the addition of the substrate (kynuramine 80 and 50 μM, for MAO-A and -B, respectively) and incubated for 20 min at 37 °C. The enzyme reactions were terminated by the addition of 78 μL of 2N NaOH to each well. The formation of 4-hydroxyquinoline (the enzyme reaction end product) was recorded fluorometrically on a SpectraMax M5 fluorescence plate reader (Molecular Devices, Sunnyvale, CA, USA) with 320 nm excitation and 380 nm emission wavelengths, using the Soft MaxPro-6 program. The inhibition of enzyme activity was calculated in terms of product formation as a percentage of corresponding controls (enzyme–substrate reaction without inhibitors). Assay controls, to define the interference of the test compounds with the fluorescence measurements, were set up simultaneously, and the enzyme or substrate was added after stopping the reaction.

### 4.3. Determination of IC_50_ Values

The enzyme assays were performed at a steady-state fixed concentration of the substrate kynuramine (80 μM for MAO-A and 50 μM for MAO-B) and different concentrations of the test compounds (PQ and NPC1161 enantiomers). The dose–response enzyme-inhibition curves were generated using Microsoft^®^ Excel (Figure 2), and the IC_50_ values were computed with XLfit^®^.

### 4.4. Enzyme Kinetics and Mechanism Studies

For determination of the binding affinity of the inhibitor (Ki) to MAO-A and -B, the enzyme assays were carried out at different concentrations of kynuramine substrate (1.90 μM to 500 μM) and at least two fixed concentrations (one each above and below the IC_50_) of the inhibitors. NPC1161A was tested at 5 and 10 μM for both MAO-A and -B. NPC1161B was tested at 7.5 and 15 μM for both MAO-A and -B. The control set without inhibitor was also run simultaneously. The results were analyzed in SigmaPlot version 10 using standard double reciprocal Lineweaver–Burk plots for computing Km and Vmax values, which were further analyzed to determine the Ki values [63].

### 4.5. Analysis of Binding of Inhibitors with the Enzymes

Enzyme-inhibitors mostly produce inhibition of the target enzyme through the formation of an enzyme–inhibitor complex. Formation of the enzyme–inhibitor complex may be accelerated in the presence of a high concentration of the test inhibitor. The property of binding of each test compound to MAO-A or -B was determined by the formation of the enzyme–inhibitor complex during incubation of the enzyme with a high concentration of the test compound. The inhibitors at about 10XIC50 concentrations were incubated with high concentrations of the enzyme. This was followed by extensive equilibrium dialysis of the enzyme–inhibitor complex. Recovery of catalytic activity of MAO-A and -B was determined before and after the dialysis. The *r*hMAO-A or -B enzyme (0.2 mg/mL protein) was incubated with each test compound in 1 mL of potassium phosphate buffer (100 mM, pH 7.4). After 20 min incubation at 37 °C, the reaction was stopped by chilling the tubes in an ice bath. All the samples with enzyme–inhibitor complex were individually dialyzed against potassium phosphate buffer (25 mM; pH 7.4) at 4 °C for 16–18 h (including three buffer changes). The control enzyme (without inhibitor) was also run through the same procedure and the activity of the enzyme was determined before and after the dialysis [63].

### 4.6. Computational Docking Methods for PQ, NPC1161 and Their Enantiomers with MAO-A and -B

The docking calculations were performed using the Glide software [71]. The MAO-A and -B crystal structures were obtained from the Protein Data Bank with the accession codes 2Z5X for MAO-A (human MAO-A complexed with harmine at 2.2 Å resolution) [72] and 1OJ9 for MAO-B (human mitochondrial MAO-B complexed with isatin at 1.7 Å resolution) [73]. These two crystal structures have been used in several previous docking studies on the binding of various ligands with MAO-A and MAO-B [74,75,76,77]. In particular, it was found that 1OJ9 had the best self-docking performance compared to other MAO-B crystal structures [77]. Prior to docking, all the water molecules in 2Z5X and 1OJ9 were removed and N5 of FAD was set as the central point of the active site for Glide. To prepare the ligands, conformational searches were performed on each ligand using the MacroModel program (MacroModel 2011). The OPLS2005 force field was employed and all the conformers within a relative energy of 50 kJ mol^–1^ were docked into MAO-A and -B using extra precision (XP) docking. The XP module in Glide was also used to rank the obtained binding poses. Unless otherwise noted, the best poses as ranked by Glide are the ones presented in the results and associated figures.

## 5. Conclusions

Primaquine (PQ), the prototype racemic 8-aminoquinoline, and NPC1161, a potent antimalarial from the class, both show enantioselective interactions with human monoamine oxidase (MAO)-A and -B. Individual enantiomers of PQ and NPC1161 were tested for in vitro inhibition, binding and computational docking with MAO-A and -B. The *R*-(-)PQ shows stronger interactions with MAO-A compared to MAO-B, while *S*-(+)PQ showed stronger interaction with MAO-B compared to MAO-A. Both *S* and *R* enantiomers of NPC1161 (NPC1161A and NPC1161B) showed more prominent inhibition of MAO-B compared to A, with about 3- and 10-fold selectivity, respectively. Differential interactions of PQ and NPC1161 enantiomers with human MAOs may be partly responsible for the enantioselective pharmacokinetic, pharmacological and toxicological properties of these anti-infective 8-AQs therapeutics. Selective and potent inhibition of MAO-B by the NPC1161A enantiomer may have potential neuropharmacological actions and provide an additional justification for further clinical advancement of NPC1161B enantiomer, which shows relatively moderate interaction with human monoamine oxidases.

## Figures and Tables

**Table 1:** Inhibition of MAO-A and MAO-B with primaquine and NPC1161 racemate and enantiomers at steady-state enzyme kinetics. The results are presented as mean ± SD of at least three observations.

8-Aminoquinolines	MAO -A IC50 (µM) ± SD	MAO -B IC50 (µM) ± SD
(*RS*)-(±)-Primaquine	87.83. ± 3.049	106.75 ± 1.181
(*S*)-(+)-Primaquine	>200.00	170.00 ± 15.27
(*R*)-(−)-Primaquine	74.33 ± 2.96	116.66 ± 8.819
(*RS*)-(±)-NPC1161	6.18 ± 0.361	1.68 ± 0.024
(*S*)-(+)-NPC1161A	5.24 ± 0.112	0.54 ± 0.012
(*R*)-(−)-NPC1161B	9.70 ± 0.178	2.93 ± 0.017
Harmine	0.0051 ± 0.0001	53.08 ± 3.915
Safinamide	88.00 ± 1.414	0.058 ± 0.002
Clorgyline [63]	0.004 ± 0.0005	-
Deprenyl [63]	-	0.49 ± 0.0036

^1^ The IC_50_ values computed from the concentration-response inhibition curves are mean ± SD of triplicate observations. Clorgyline (a selective MAO-A inhibitor) and deprenyl (a selective MAO-B inhibitor) were tested simultaneously as reference standards.

**Table 2:** Determination of Ki values for binding/inhibition of human MAO-A and -B by enantiomers of NPC1161.

Compound	MAO-A		MAO-B	
	Ki (µM) ± SD	Type of Inhibition	Ki (µM) ± SD	Type of Inhibition
(*S*)-(+)-NPC1161A	3.298± 0.508	Mixed type/ irreversible	0.294 ± 0.080	Mixed type/ irreversible
(*R*)-(−)-NPC1161B	3.993± 0.640	Mixed type/ reversible	1.385± 0.284	Mixed type/ reversible

Values are mean ± SD of triplicate experiments.

**Table 3:** Determination of Ki values for binding/inhibition of human MAO-A and -B by enantiomers of NPC1161.

Compound	Docking Scores	
	MAO-A	MAO-B
(*S*)-(+)-PQ	−8.523	−8.220
(*R*)-(−)-PQ	−8.822	−8.565
(*S*)-(+)-NPC1161A	−8.983	−9.518
(*R*)-(−)-NPC1161B	−8.968	−9.234

## Data Availability

The generated datasets and/or analyzed data during the research study available from the corresponding author on request.
